# Comparison of Ultra-Wide Field Photography to Ultra-Wide Field Angiography for the Staging of Sickle Cell Retinopathy

**DOI:** 10.3390/jcm11040936

**Published:** 2022-02-11

**Authors:** Héloise Torres-Villaros, Franck Fajnkuchen, Fatima Amari, Lucie Janicot, Audrey Giocanti-Aurégan

**Affiliations:** 1 Ophthalmology Department, Hôpital Avicenne, 93000 Bobigny, France; heloise.torres.villaros@gmail.com (H.T.-V.); franck.fajnkuchen@avc.aphp.fr (F.F.); fatima.amari@aphp.fr (F.A.); lucie.janicot@aphp.fr (L.J.); 2 Centre D’Imagerie et de Laser, 75015 Paris, France

**Keywords:** sickle cell retinopathy, Goldberg classification, ultra-wide field imaging, ultra-wide field florescin angiography

## Abstract

Sickle cell retinopathy (SCR) is classified by Goldberg based on peripheral vascular changes. Ultra-wide field (UWF) imaging has enhanced visualization of the peripheral retina. However, there is no consensus on the optimal imaging technique for the screening of SCR. We performed a monocentric observational cross-sectional study to compare UWF fundus photography (UWF-FP) with UWF angiography (UWF-FA). All patients who underwent UWF-imaging (Optos, PLC, Scotland, UK) for screening of sickle cell retinopathy between January 2016 and December 2019 were retrospectively included. Eyes with previous laser treatment or concomitant retinal disease were excluded. UWF-FP images were graded based on the Goldberg classification by four graders with various degrees of experience. UWF-FA pictures were reviewed by an independent retina specialist. Differences in Goldberg staging across UWF-FP and UWF-FA were assessed. A total of 84 eyes of 44 patients were included. Based on UWF-FA, most eyes were stage 2 (77.4%) and 19 were stage 3 (22.6%). The pre-retinal neovascularization detection sensitivity on UWF-FP was 52.6 to 78.9%, depending on the graders. UWF-FA led to a later Goldberg stage of retinopathy, in most cases from stage 1 to stage 2. Neovascularization (stage 3) was not detected by our graders on UWF-FP in 21.1 to 57.9% of eyes. UWP-FP tends to underestimate Goldberg stages of retinopathy compared with UWF-FA and is less accurate when detecting neovascularization in sickle cell retinopathy, which has a direct impact on therapeutic management and prognosis.

## 1. Introduction

Sickle cell disease (SCD) is one of the most common, worldwide monogenetic disorders caused by mutations in the β-globin gene. Vaso-occlusive complications affect almost all ocular structures, but sickle cell retinopathy (SCR) remains the most important in terms of frequency and visual impairment. Chronic retinal vascular ischemia secondary to rigid sickle-shaped red blood cells stimulate the production of proangiogenic factors such as vascular endothelial growth factor (VEGF) which can lead to pathologic retinal neovascularization [1,2]. The clinical course of sickle cell retinopathy is variable and dependent on different genotypes. It is known that hemoglobin SS patients are less likely to develop advanced stage retinopathy than patients with hemoglobin SC and other variant genotypes. Proliferative sickle cell retinopathy is reported to affect 3.5–30.2% of hemoglobin SS patients and 25.3–75.9% of hemoglobin SC patients [3,4,5,6,7,8,9].

Since patients remain mostly asymptomatic until vision-threatening complications arise, a periodic fundus examination is recommended. However, there is no consensus in recommendations on the optimal imaging by which to evaluate retinopathy in SCD patients. Goldberg’s classification remains the most widely used staging method, based on features observed during clinical examination and 30-degree standard fluorescein angiography (FA) [10]. Since Goldberg’s publication, novel ultra-wide field (UWF) imaging systems have greatly enhanced our ability to observe vascular remodeling occurring in the far retinal periphery where sickle cell retinopathy is often first detectable. Ultra-wide field fundus photography (UWF-FP) and ultra-wide field fluorescein angiography (UWF-FA) provide a visualization of up to 200° of the retina captured in a single frame. However, UWF-FA remains an invasive procedure with potentially severe side effects.

The aim of this study was to assess the diagnostic performance of UWF-FP for the detection of SCR with UWF-FA as the reference exam and determine if UWF-FP is a valuable exam for the screening of SCD patients.

## 2. Materials and Methods

This cross-sectional monocentric study was conducted at Avicenne Hospital, Bobigny, France, a center specialized in retinal disease imaging and treatment. All consecutive patients with genetically confirmed SCD referred for systematic ophthalmological examination between January 2016 and December 2019 were included. Patients with other confounding retinal vascular disease, eyes with previous laser treatment for sickle cell retinopathy or other vascular retinopathies were excluded. Each patient underwent ultra-wide field fundus photography (UWF-FP) and an ultra-wide field fluorescein angiography (UWF-FA), mostly on the same day or within 3 months at most. UWF-FP and UWF-FA were performed using Optos 200 Tx (Optos PLC, Dunfermline, Scotland, UK). Eyes with low image quality and/or significant media opacity precluding accurate interpretation of UWF imaging were excluded. Data on age, sex and genotype of SCD were recorded from each patient. The study was conducted in accordance with the Declaration of Helsinki. Informed consent was obtained from all subjects involved in the study.

UWF-FP pictures were independently analyzed by two retina specialists and two ophthalmology residents blinded to patient information. The graders could adjust magnification, brightness, filters and contrast of the images on the Optos viewer screen, whereas all other additional information was hidden. Eyes were staged according to Goldberg scale for proliferative sickle retinopathy [10]: peripheral arterial occlusions (stage 1), arteriovenous anastomoses (stage 2), neovascularization (stage 3), vitreous hemorrhage (stage 4), retinal detachment (stage 5). Eyes with no retinopathy were noted as Goldberg stage 0. In addition to clinician grading, the UWF-FA images were reviewed by a third independent retina specialist (AGA) and graded based on the Goldberg classification.

Statistical analysis was performed using Xlstat software (version 2020, Addinsoft, Paris, France). A Chi-squared test was used to assess the relationship between severity of retinopathy and genotype. A *p* value ≤ 0.05 was considered significant. Sensitivity and specificity of UWF-FP were calculated on the basis of the neovascular status using UWF-FA as the gold standard. Percentage agreement between masked graders, as well as Fleiss’ Kappa statistic, was calculated to assess inter-grader agreement.

## 3. Results

In total, 84 eyes of 44 patients (22 males and 22 females), mean age 33 ± 10.4 years, were included in the study. A total of 4 out of 88 eyes were excluded because of pre-existing sector retinal photocoagulation. Sickle genotypes included 21 patients with sickle SS (47.7%), 17 with SC (38.6%), and 6 with β + thalassemia (13.6%).

Table 1 summarizes the results for Goldberg staging based on UWF-FA imaging and stratified by genotype within the study cohort. Most eyes were Goldberg stage 2 (76.2%). 19 eyes (22.6%) demonstrated neovascularization (stage 3). No eyes with vitreous hemorrhage (stage 4) or retinal detachment (stage 5) were included. A total of 5 (26%) of the 14 patients grading stage 3 had bilateral proliferative lesions. When stratified by genotype, eyes from patients with sickle SC tended to be at a later stage, with more eyes being at Goldberg stage 3 (29%) than eyes with sickle SS (19%), but the difference was not significant (*p* = 0.32).

The change in Goldberg staging across UWF-FP and UWF-FA for each grader is summarized in Table 2. Correct classification of UWF-FP was performed in 63.1% (53 eyes) and 76.2% (64 eyes) of eyes studied by the two retina specialists, and 52.4% (44 eyes) and 65.5% (55 eyes) of eyes when studied by the two residents. Most of the disagreements involved a discrepancy between Goldberg stage 1 or 2 (Table 3).

Sensitivity and specificity of UWP-FP for the detection of proliferative SCD is detailed in Table 4. Sensitivity was 65.8% (95% confidence interval [CI95%] 49.9–78.8) for the retina specialists and 57.9% ([CI95%] 42.2–72.1) for the ophthalmology residents. Specificity was 86.4% ([CI95%] 79.8–91.1) for the retina specialists and 99.2% ([CI95%] 95.8–99.9) for the ophthalmology residents. The retina specialist with the highest sensitivity had the lowest specificity since 15 eyes were graded as a stage 3 by mistake. Reading time of all images is indicated in Table 4 and ranged from 195 to 340 min, depending on the graders.

Intergrader agreement for the detection of neovascular lesions on UWF-FP was poor with a kappa coefficient of 0.33. In addition, a lower kappa coefficient of 0.17 was seen when comparing Goldberg staging on UWF-FP.

## 4. Discussion

Sickle cell retinopathy is classified based on peripheral vascular changes. Since the development of the Goldberg classification system, ultra-wide field imaging has increased our understanding of SCR through greater visualization of the peripheral retina. Despite these advances, limited literature exists on the topic of UWF imaging for detection of SCR and its implications for monitoring SCD patients. Although very useful, UWF-FA carries some drawbacks similar to that of standard fluorescein angiography including the cost of the procedure, time burden on the patient and potential adverse events. The aim of our study was to evaluate the value of UWF-FP alone when used to screen peripheral retinal changes in SCD patients.

Published reports on the use of UWF imaging for a range of peripheral retinopathies including diabetic retinopathy [11,12,13,14,15], vein occlusion [16,17], and retinopathy of prematurity [18] support the clinical benefit. Similarly, earlier studies confirm the advantages of UWF imaging in detecting more sickle cell induced retinal microvascular abnormalities than traditional non-widefield imaging [19,20,21,22]. The first report which compared UWF-imaging with classical ETDR-7-field images was published by Cho et al. in 2011, who evaluated 12 eyes of 6 sickle cell disease patients [19]. The study revealed that UWF-FA identified peripheral microvascular abnormalities undetected by seven-standard field FA in 91.7% (11/12) eyes studied. Furthermore, in 25% (3/12) of eyes UWF-FA detected neovascularization missed by clinical examination. Of the three eyes, two underwent treatment with sector retinal photocoagulation. This study was limited by its small sample size. These results were supported by a later, larger study from our team, evaluating 59 eyes of 30 patients by two ophthalmologist graders [20]. UWF-imaging revealed a more severe Goldberg classification in 88.1% of eyes than seven standard field montages. 82.7% (24/29) of eyes with preretinal neovascularization diagnosed based on both UWF-FP and UWF-FA were missed when ETDRS-7-field images were used.

**Figure 1:**
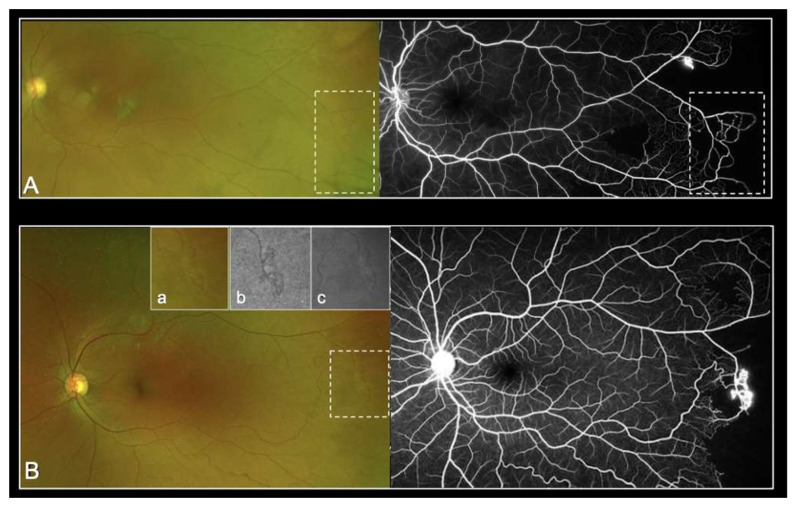
Representative UWF-imaging with peripheral arteriovenous anastomoses (stage 2 Goldberg) (**A**) and with sea fan lesion (stage 3 Goldberg) (**B**) clearly visible in color photography (**a**), red-free filter (**b**) and autofluorescence (**c**).

As demonstrated in Figure 1, UWP-FP allowed identifiable visualization of sickle cell manifestations in the many eyes studied. The use of additional red-free and autofluorescence pictures also helped identify vascular abnormalities.

**Figure 2:**
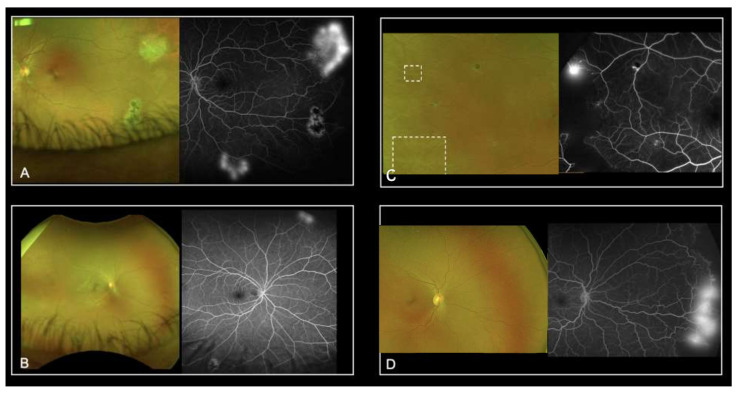
Representative UWF-imaging where neovascularization was not detected on the UWF-FP: sea fan lesion was masked by eyelids (**A**), it was out of the field of view and revealed by leakage on the late frames of UWF-FA (**B**) or resolution was insufficient (**C**,**D**).

**Figure 3:**
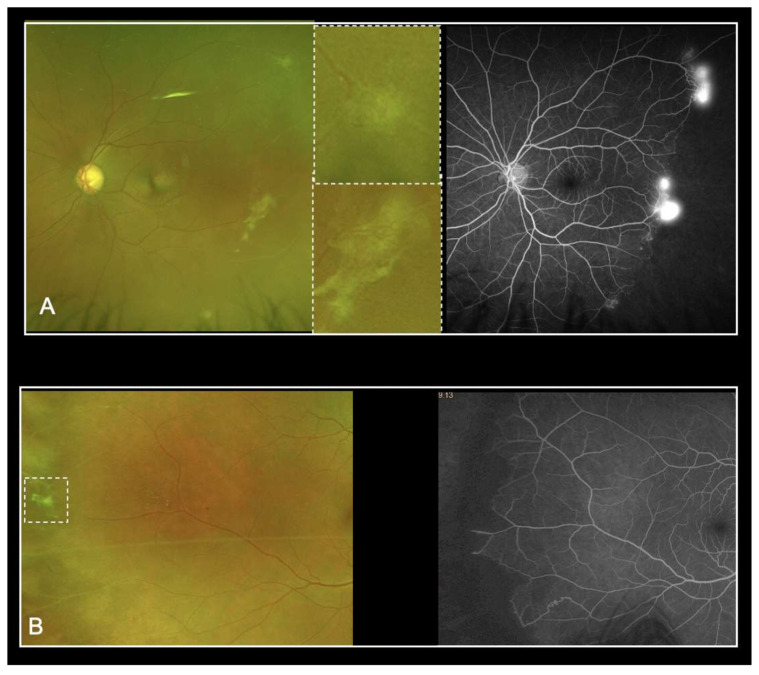
Sea fan lesions associated with retina fibrosis on UWF-FP: UWF-FA revealed persistent leakage (**A**) or complete sea fan fibrosis with no leakage (**B**).

Despite the previously described advantages of UWF-imaging, our study revealed severe limitations by using UWF-FP alone for the screening of SCD patients. The Goldberg staging based on UWF-FP was efficient in only 52.6% to 78.9% of eyes studied, depending on the graders. Grading time was highly variable among the graders, which may explain in part the variability of results. Additionally, UWF-FA led to a higher perceived Goldberg stage of retinopathy. In most cases, the staging changed from Goldberg stage 1 to stage 2. These results highlight the difficulty in visualizing peripheral anastomoses on UWF-FP. Moreover, UWF-FA showed neovascularization not detected on UWF-FP from 21.1% and up to 57.9% of eyes. Amongst all the graders, the sensitivity of UWF-FP was not acceptable. We retrospectively identified four types of mistake (Figure 2). Firstly, several sea fan lesions were subtle and almost undetectable on the UWF-FP pictures, even on the red-free and autofluorescence images. This could be explained in part by the peripheral distortion and decreased resolution of the far peripheral retina on Optos. Secondly, some neovascular lesions were too peripheral and invisible on the 200° UWF-FP pictures, equating to 82% of the total retinal surface area. Leakage on the late frames of angiography facilitated their detection. Thirdly, sea fan lesions were sometimes associated with retinal fibrosis and UWP-FP alone did not predict neovascular activity. Although they had a similar clinical aspect on UWF-FP, UWF-FA showed partial fibrosis with leakage for some, and complete sea fan fibrosis with no leakage for others (Figure 3). Prognosis varies since progression to stage 4 or 5 is more frequent in cases of persistent leakage [23]. Fourthly, some microvascular abnormalities were confounded with neovascular lesions. Interpretation was distorted by Optos limitations including abnormal colors and peripheral distortions. Furthermore, eyelid artifacts were a recurrent issue and graders were granted the time they needed to screen pictures, which did not reflect realistic time allowances during clinical practice. We then hypothesized that our study underestimates the misdiagnosis of neovascular lesions based on UWF-FP imaging.

Although UWF Optos has allowed better analysis of the retinal periphery, there are still some limitations that may partly explain our difficulties in detecting lesions of SCR [24]. The wide-field view of Optomap was achieved by employing a scanning laser ophthalmoscope while utilizing an ellipsoid mirror to obtain images of the retinal periphery. It uses a red (633 nm) and a green (523 nm) laser that produce a pseudo-color retinal image, which differs from the true color image produced by traditional fundus cameras using a white-light source. Optomap images represent a compromise between the surface area of retina captured and resolution required to see relevant retinal changes and so there is a decreased resolution when compared with other color fundus imaging modalities. Peripheral distortion occurs because of the Optomap using an ellipsoid mirror. Distortion and decreased resolution are more pronounced in the nasal and temporal far periphery. There is also a variability in the total retinal surface captured between images or imaging sessions that can be attributed to the presence of image artefacts.

Our findings contrast with earlier studies. Han et al. evaluated 70 eyes of 35 patients and showed that UWF-FA did not change treatment decisions compared with ophthalmoscopy and UWF-FP [25]. Though UWF imaging showed greater severity, most clinical gradings only increased from Goldberg stage 1 to Goldberg stage 2, both of which are non-proliferative stages and do not vary in therapeutic management. In only one eye UWF-FA revealed neovascularization undetected by the clinician or UWF-FP, and in this case the lesion was not deemed severe enough to indicate photocoagulation treatment. The small number of participants with proliferative disease (8 of 70 eyes) included in this study may have skewed results. These results were supported by a later report which evaluated the diagnostic performance of UWF-FP with UWP-FA using Optos as the reference exam in 66 eyes (33 patients) graded by two masked ophthalmologists [26]. The sensitivity of UWP-FP alone for the detection of neovascularization was 100% for both graders and the specificity was 100% for the retina specialist, and 98.1% for the ophthalmology resident. Similarly, UWF-FP tends to underestimate the Goldberg stage compared with UWF-FA, predominantly for stages 1 and 2. The two graders in this study assessed the Goldberg stage both on UWF-FP and UWP-FA, whereas the four graders in our analysis were blinded to angiography to determine the stage using only UWF-FP. This may partly explain the discrepancies between our results.

For diabetic retinopathy as well, it has been shown that there are discrepancies in severity scores between color fundus photography and fluorescein angiography among eyes treated by anti-vascular endothelial growth factor (anti-VEGF) therapy. Anti-VEGF may indeed improve the diabetic retinopathy severity score on UWF-FP by clearing the indirect sign of ischemia, without changing the retinal non-perfusion on UWF-FA [27].

The main limitation of our study was the small number of graders, which explained in part the variability of UWF-FP interpretation between readers with various levels of experience. Due to the rare occurrence of sea fan lesions, the 95% CI of the sensitivity and specificity varied greatly. Further prospective and larger studies are needed to confirm UWF-FA as the gold standard exam for monitoring SCR.

Our study confirms that UWF-FP tends to underestimate Goldberg stages compared with UWF-FA. Moreover, in contrast of earlier reports, UWF-FP imaging appears to be less accurate and sensitive than UWF-FA in detecting neovascularization. These findings directly impact therapeutic management, as stage 3 corresponds to the classical indication for retinal photocoagulation treatment [28]. Although UWF-FP appears to be a helpful tool with which to highlight sickle cell retinopathy manifestations, our results suggest that UWF-FA is more sensitive to neovascularization detection and more accurate in the diagnosis of SCR, allowing a great view of non-perfused retinae, arteriovenous anastomoses, and leakages associated with neovascularization.

## Figures and Tables

**Table 1:** Goldberg staging based on UWF-FA and stratified by genotype.

	All Genotypes Eyes (%)	SS Eyes (%)	SC Eyes (%)	β-Thal Eyes (%)
Stage 0	0 (0)	0 (0)	0 (0)	0 (0)
Stage 1	1 (1.2)	0 (0)	1 (3.2)	0 (0)
Stage 2	64 (76.2)	34 (81)	21 (67.7)	9 (81.9)
Stage 3	19 (22.6)	8 (19)	9 (29)	2 (18.1)
Total	84 (100)	42 (50)	31(36.9)	11 (9.1)

**Table 2:** Differences in Goldberg staging across UWF-FP and UWF-FA.

Stages Difference UWF-FP vs. UWF-FA	Retina Specialists (Eyes)		Ophthalmologist Residents (Eyes)	
	Grader 1	Grader 2	Grader 1	Grader 2
−2 stage difference	14	0	6	5
−1 stage difference	12	5	22	35
No stage difference	53	64	55	44
+1 stage difference	5	14	1	0
+2 stage difference	0	0	0	0

**Table 3:** Disagreements in Goldberg staging based on UWF-FP examination.

UWF-FA	UWF-FP	Retina Specialists (Eyes)		Ophthalmology Residents (Eyes)		Total Eyes (%)
		Grader 1	Grader 2	Grader 1	Grader 2	
Stage 1	Stage 3	-	1	-	-	1 (0.8)
Stage 2	Stage 0	10	0	5	4	19 (15.8)
	Stage 1	7	1	18	25	51 (42.5)
	Stage 3	5	14	1	0	20 (16.7)
Stage 3	Stage 1	4	0	1	1	6 (5.0)
	Stage 2	5	4	4	10	23 (19.2)

**Table 4:** Sensitivity and specificity of UWF-FP for detection of neovascular lesions (*n* = 19 eyes in stage 3) and reading time of 84 images per grader.

	Sensitivity [CI95%]	Specificity [CI95%]	Reading Time (min)
All readers	61.8% [50.6–71.9]	92.3% [88.4–94.9]	
Retina specialists	65.8% [49.9–78.8]	86.4% [79.8–91.1]	
Grader 1	52.6% [31.7–72.7]	92.3% [83.2–98.7]	340
Grader 2	78.9% [56.7–91.5]	78.5% [67.0–86.8]	280
Ophthalmologist residents	57.9% [42.2–72.1]	99.2% [95.8–99.9]	
Grader 1	73.7% [51.2–88.2]	98.5% [91.8–99.7]	300
Grader 2	42.1% [23.1–63.7]	100% [94.4–1]	195

## Data Availability

Data are available upon request at heloise.torres.villaros@gmail.com.

## References

[1] Cao J., Mathews M.K., McLeod D.S., Merges C., Hjelmeland L.M., Lutty G.A. (1999). Angiogenic Factors in Human Proliferative Sickle Cell Retinopathy. Br. J. Ophthalmol..

[2] Rodrigues M., Kashiwabuchi F., Deshpande M., Jee K., Goldberg M.F., Lutty G., Semenza G.L., Montaner S., Sodhi A. (2016). Expression Pattern of HIF-1α and VEGF Supports Circumferential Application of Scatter Laser for Proliferative Sickle Retinopathy. Investig. Ophthalmol. Vis. Sci..

[3] Leveziel N., Bastuji-Garin S., Lalloum F., Querques G., Benlian P., Binaghi M., Coscas G., Soubrane G., Bachir D., Galactéros F. (2011). Clinical and Laboratory Factors Associated with the Severity of Proliferative Sickle Cell Retinopathy in Patients with Sickle Cell Hemoglobin C (SC) and Homozygous Sickle Cell (SS) Disease. Medicine.

[4] Diallo J.W., Sanfo O., Blot I., Meda N., Sawadogo P., Ouedraogo A., Simporé J. (2009). Étude Épidémiologique et Facteurs Pronostiques de La Rétinopathie Drépanocytaire à Ouagadougou (Burkina Faso). J. Français D’ophtalmol..

[5] Downes S.M., Hambleton I.R., Chuang E.L., Lois N., Serjeant G.R., Bird A.C. (2005). Incidence and Natural History of Proliferative Sickle Cell Retinopathy. Ophthalmology.

[6] Dembélé A.K., Toure B.A., Sarro Y.S., Guindo A., Fané B., Offredo L., Kené S., Conaré I., Tessougué O., Traoré Y. (2017). Prévalence et Facteurs de Risque de La Rétinopathie Drépanocytaire Dans Un Centre de Suivi Drépanocytaire d’Afrique Subsaharienne. La Rev. Méd. Interne.

[7] Leveziel N., Lalloum F., Bastuji-Garin S., Binaghi M., Bachir D., Galacteros F., Souied E. (2012). Rétinopathie Drépanocytaire: Analyse Rétrospective Portant Sur 730 Patients Suivis Dans Un Centre de Référence. J. Français d’Ophtalmol..

[8] Fox P.D., Dunn D.T., Morris J.S., Serjeant G.R. (1990). Risk Factors for Proliferative Sickle Retinopathy. Br. J. Ophthalmol..

[9] Clarkson J.G. (1992). The Ocular Manifestations of Sickle-Cell Disease: A Prevalence and Natural History Study. Trans. Am. Ophthalmol. Soc..

[10] Goldberg M.F. (1971). Classification and Pathogenesis of Proliferative Sickle Retinopathy. Am. J. Ophthalmol..

[11] Rasmussen M.L., Broe R., Frydkjaer-Olsen U., Olsen B.S., Mortensen H.B., Peto T., Grauslund J. (2015). Comparison between Early Treatment Diabetic Retinopathy Study 7-Field Retinal Photos and Non-Mydriatic, Mydriatic and Mydriatic Steered Widefield Scanning Laser Ophthalmoscopy for Assessment of Diabetic Retinopathy. J. Diabetes Its Complicat..

[12] Silva P.S., Cavallerano J.D., Sun J.K., Soliman A.Z., Aiello L.M., Aiello L.P. (2013). Peripheral Lesions Identified by Mydriatic Ultrawide Field Imaging: Distribution and Potential Impact on Diabetic Retinopathy Severity. Ophthalmology.

[13] Oliver S.C.N., Schwartz S.D. (2010). Peripheral Vessel Leakage (PVL): A New Angiographic Finding in Diabetic Retinopathy Identified with Ultra Wide-Field Fluorescein Angiography. Semin. Ophthalmol..

[14] Wessel M.M., Aaker G.D., Parlitsis G., Cho M., D’Amico D.J., Kiss S. (2012). Ultra-Wide-Field Angiography Improves the Detection and Classification of Diabetic Retinopathy. Retina.

[15] Friberg T.R., Gupta A., Yu J., Huang L., Suner I., Puliafito C.A., Schwartz S.D. (2008). Ultrawide Angle Fluorescein Angiographic Imaging: A Comparison to Conventional Digital Acquisition Systems. Ophthalmic Surg. Lasers Imaging Retin..

[16] Spaide R.F. (2011). Peripheral Areas of Nonperfusion in Treated Central Retinal Vein Occlusion as Imaged by Wide-Field Fluorescein Angiography. Retina.

[17] Prasad P.S., Oliver S.C.N., Coffee R.E., Hubschman J.-P., Schwartz S.D. (2010). Ultra Wide-Field Angiographic Characteristics of Branch Retinal and Hemicentral Retinal Vein Occlusion. Ophthalmology.

[18] Patel C.K., Fung T.H.M., Muqit M.M.K., Mordant D.J., Brett J., Smith L., Adams E. (2013). Non-Contact Ultra-Widefield Imaging of Retinopathy of Prematurity Using the Optos Dual Wavelength Scanning Laser Ophthalmoscope. Eye.

[19] Cho M., Kiss S. (2011). Detection and Monitoring of Sickle Cell Retinopathy Using Ultra Wide-Field Color Photography and Fluorescein Angiography. Retina.

[20] Drouglazet G., Fajnkuchen F., Amari F., Bodaghi B., Giocanti-Aurégan A. (2019). Comparison between Ultra-Widefield and 7-Standard Field Angiography for Proliferative Sickle Cell Retinopathy Screening, Follow-up and Classification. J. Ophthalmol. Clin. Res..

[21] Alabduljalil T., Cheung C.S., VandenHoven C., Mackeen L.D., Kirby-Allen M., Kertes P.J., Lam W.-C. (2021). Retinal Ultra-Wide-Field Colour Imaging versus Dilated Fundus Examination to Screen for Sickle Cell Retinopathy. Br. J. Ophthalmol..

[22] Linz M.O., Scott A.W. (2019). Wide-Field Imaging of Sickle Retinopathy. Int. J. Retin. Vitr..

[23] Sayag D., Binaghi M., Souied E.H., Querques G., Galacteros F., Coscas G., Soubrane G. (2008). Retinal Photocoagulation for Proliferative Sickle Cell Retinopathy: A Prospective Clinical Trial with New Sea Fan Classification. Eur. J. Ophthalmol..

[24] Quinn N., Csincsik L., Flynn E., Curcio C.A., Kiss S., Sadda S.R., Hogg R., Peto T., Lengyel I. (2019). The Clinical Relevance of Visualising the Peripheral Retina. Prog. Retin. Eye Res..

[25] Han I.C., Zhang A.Y., Liu T.Y.A., Linz M.O., Scott A.W. (2019). Utility of Ultra-Widefield Retinal Imaging for the Staging and Management of Sickle Cell Retinopathy. Retina.

[26] Bunod R., Mouallem-Beziere A., Amoroso F., Capuano V., Bitton K., Kamami-Levy C., Jung C., Souied E.H., Miere A. (2019). Sensitivity and Specificity of Ultrawide-Field Fundus Photography for the Staging of Sickle Cell Retinopathy in Real-Life Practice at Varying Expertise Level. J. Clin. Med..

[27] Bonnin S., Dupas B., Lavia C., Erginay A., Dhundass M., Couturier A., Tadayoni R. (2019). Anti–vascular endothelial growth factor therapy can improve diabetic retinopathy score without change in retinal perfusion. Retina.

[28] Myint K.T., Sahoo S., Thein A.W., Moe S., Ni H. (2015). Laser Therapy for Retinopathy in Sickle Cell Disease. Cochrane Database Syst. Rev..

